# Shape Trumps Size: Image-Based Morphological Analysis Reveals That the 3D Shape Discriminates Intracranial Aneurysm Disease Status Better Than Aneurysm Size

**DOI:** 10.3389/fneur.2022.809391

**Published:** 2022-05-03

**Authors:** Norman Juchler, Sabine Schilling, Philippe Bijlenga, Vartan Kurtcuoglu, Sven Hirsch

**Affiliations:** ^1^School of Life Sciences and Facility Management, Institute of Computational Life Sciences, Zurich University of Applied Sciences, Wädenswil, Switzerland; ^2^The Interface Group, Institute of Physiology, University of Zurich, Zurich, Switzerland; ^3^Lucerne School of Business, Institute of Tourism and Mobility, Lucerne University of Applied Sciences and Arts, Lucerne, Switzerland; ^4^Neurosurgery Division, Department of Clinical Neurosciences, Geneva University Hospital and Faculty of Medicine, Geneva, Switzerland; ^5^Zurich Center for Integrative Human Physiology, University of Zurich, Zurich, Switzerland; ^6^National Center of Competence in Research, Kidney.CH, Zurich, Switzerland; ^7^Neuroscience Center Zurich, University of Zurich, Zurich, Switzerland

**Keywords:** intracranial aneurysms, image-based analysis, rupture status prediction, quantitative morphology, shape irregularity

## Abstract

**Background:**

To date, it remains difficult for clinicians to reliably assess the disease status of intracranial aneurysms. As an aneurysm's 3D shape is strongly dependent on the underlying formation processes, it is believed that the presence of certain shape features mirrors the disease status of the aneurysm wall. Currently, clinicians associate irregular shape with wall instability. However, no consensus exists about which shape features reliably predict instability. In this study, we present a benchmark to identify shape features providing the highest predictive power for aneurysm rupture status.

**Methods:**

3D models of aneurysms were extracted from medical imaging data (3D rotational angiographies) using a standardized protocol. For these aneurysm models, we calculated a set of metrics characterizing the 3D shape: Geometry indices (such as undulation, ellipticity and non-sphericity); writhe- and curvature-based metrics; as well as indices based on Zernike moments. Using statistical learning methods, we investigated the association between shape features and aneurysm disease status. This processing was applied to a clinical dataset of 750 aneurysms (261 ruptured, 474 unruptured) registered in the AneuX morphology database. We report here statistical performance metrics [including the area under curve (AUC)] for morphometric models to discriminate between ruptured and unruptured aneurysms.

**Results:**

The non-sphericity index *NSI* (*AUC* = 0.80), normalized Zernike energies $Z_{N}^{surf}$ (*AUC* = 0.80) and the modified writhe-index ${\bar{W}}_{mean}^{L_{1}}$ (*AUC* = 0.78) exhibited the strongest association with rupture status. The combination of predictors further improved the predictive performance (without location: *AUC* = 0.82, with location *AUC* = 0.87). The anatomical location was a good predictor for rupture status on its own (*AUC* = 0.78). Different protocols to isolate the aneurysm dome did not affect the prediction performance. We identified problems regarding generalizability if trained models are applied to datasets with different selection biases.

**Conclusions:**

Morphology provided a clear indication of the aneurysm disease status, with parameters measuring shape (especially irregularity) being better predictors than size. Quantitative measurement of shape, alone or in conjunction with information about aneurysm location, has the potential to improve the clinical assessment of intracranial aneurysms.

## Introduction

Intracranial aneurysms (IAs) have a complex pathobiology and are therefore difficult to assess clinically. Confronted with an increased rate of incidentally diagnosed unruptured IAs, clinicians are in need of a marker for disease instability to better balance the risks of rupture against the risks of treatment. This marker could ideally be acquired non-invasively in the context of routine examinations.

In this context, aneurysm shape has been proposed as a candidate for such an imaging-derived marker for several reasons. Firstly, pathophysiological evidence suggests that structural changes in the aneurysmal wall are linked to macroscopic deformations of the wall (1, 2). The presence of vasa vasorum or the formation of organized luminal thrombosis, which frequently accompany IAs and are believed to unfavorably affect disease progression, do also leave an imprint in the vascular lumen as seen in contrast enhanced imaging (1, 3, 4).

Secondly, shape can be seen as an expression of hemodynamic flow patterns. The local geometry of aneurysms governs the blood flow and the fluidic forces exerted on the vessel wall. Variations of these forces have been associated with wall damage, aneurysm initiation and growth (4–8). Shape and flow dynamics are interrelated: changes in morphology influence the flow patterns in the vicinity of the aneurysm, which in turn can stimulate wall remodeling that eventually can lead to new morphological variations (4).

Thirdly, angiographic imaging is a non- or low-invasive utility readily available in clinics. It is the primary source of information for the diagnosis and treatment of IAs. In addition to its location, the anatomical embedding and the size of an aneurysm, radiologists can also infer its shape from medical images.

This wealth of evidence is contrasted by the paucity of guidelines that address morphology quantitatively. To date, the assessment of aneurysm shape is based mainly on the subjective opinion of the clinician.

The purpose of this study is to investigate and benchmark different methods to quantify aneurysm morphology and to examine how shape relates to the disease status.

## Materials and Methods

### Imaging and Patient Data

**Figure 1:**
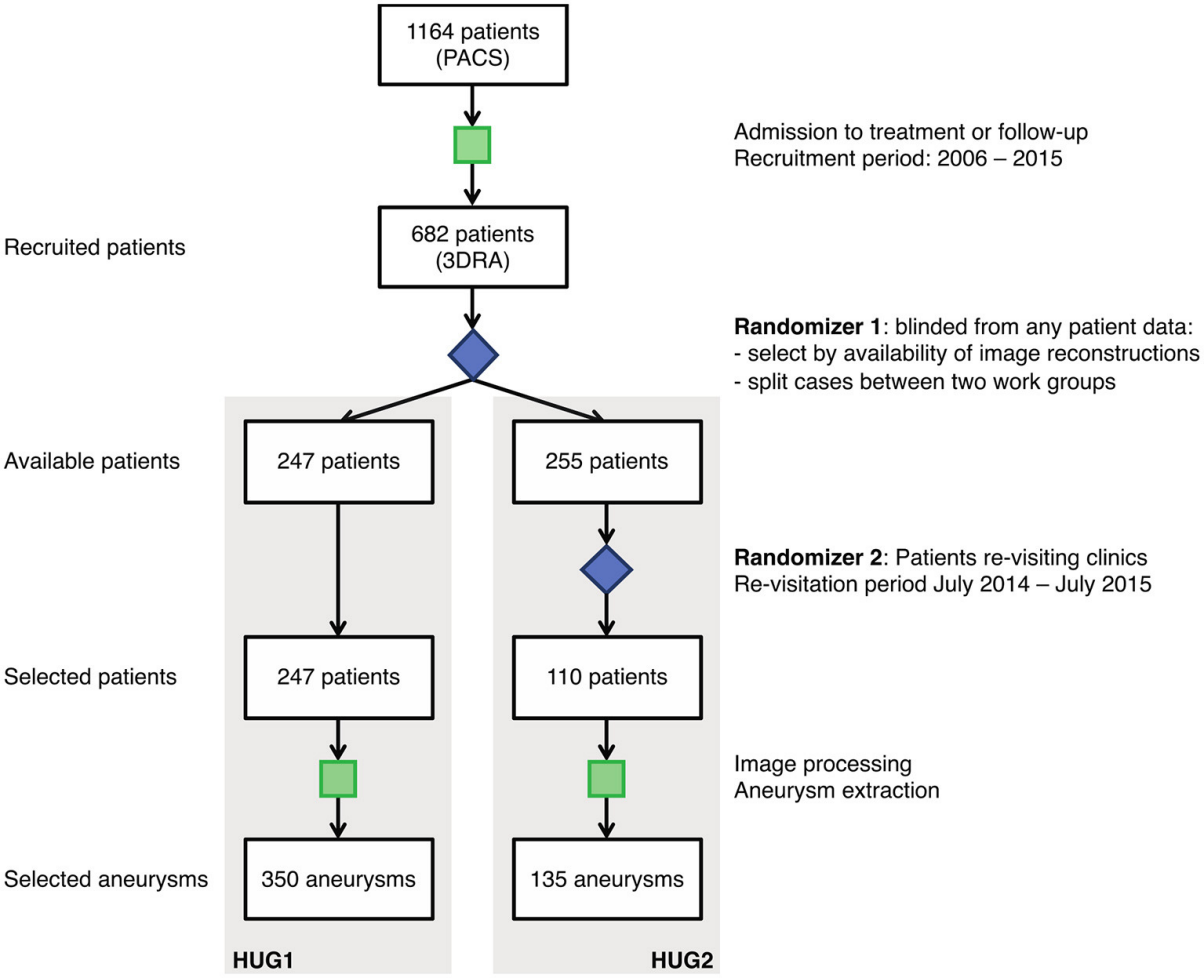
Acquisition process for the HUG dataset. Starting from the same set of recruited patients, two teams of data curators segmented the vascular structures in 3DRA images following similar protocols.

Between September 2006 and July 2015 data from 1,164 patients were collected prospectively and consecutively at the Geneva University Hospital (HUG), continuing the data collection scheme initiated and implemented during the @neurIST project (9, 10). From this initial cohort, we recruited the patients investigated by 3D rotational angiography (3DRA) because they were considered at risk for rupture or suffered from a ruptured aneurysm. A significant proportion of the cohort (41%, 482 patients) was followed up only using magnetic resonance imaging (MRI) or computed tomography (CT) imaging and was therefore not available for this study. Of the remaining 59% of the cohort (682 patients), we processed the data from 502 patients for whom image reconstructions were available. This data was split randomly between two independent teams of data curators by ignoring any prior information about the cases (Figure 1). In 180 cases no reconstructions were available for the 3DRA and therefore were excluded. While team 1 processed the 3DRA from all assigned 247 patients, team 2 selected from the total of 255 the 110 patients that visited the HUG for aneurysm repair or post-treatment follow-up examinations (scheduled 6 weeks, 3 months, 1, 2, and 5 years after treatment) during a fixed time frame of 1 year (Figure 1). Both teams processed only angiograms of aneurysms before treatment.

In addition to angiographic data, the datasets included sex, age, rupture status and anatomical location (per aneurysm) for all the cases.

To test whether our findings generalize to other datasets, we expanded the database by two external datasets: From the @neurIST project (9, 11), we included 164 aneurysms (151 patients) acquired in Barcelona, Geneva and Sheffield. From the publicly available Aneurisk database (12) we used 101 aneurysms (97 patients) retrospectively collected at the Ca' Granda Hospital, Niguarda, Milano between 2002 and 2006 (13). The data processing was described by Piccinelli et al. (14, 15).

### Data Processing

**Figure 2:**
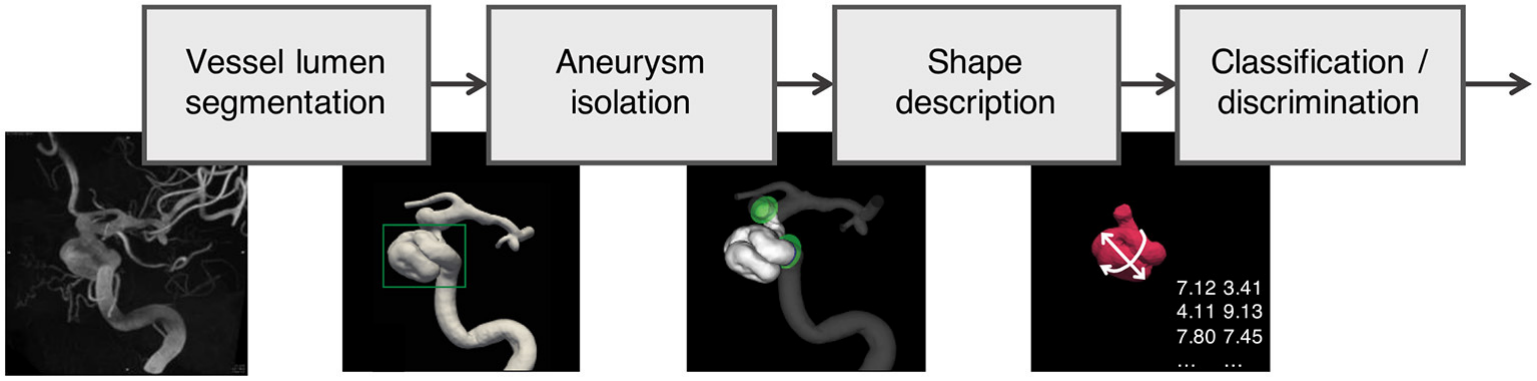
Data processing pipeline applied to all aneurysms in the AneuX morphology database: Using robust vessel lumen segmentation techniques, a geometric model of the aneurysm and the surrounding vasculature is extracted from the 3DRAs. Subsequently, the aneurysm is isolated by means of (planar or non-planar) cuts. For the resulting aneurysm models, morphometric features were computed, which were then analyzed and compared with additional clinical information about the cases (classification/discrimination). 3DRA, 3D rotational angiogram.

Figure 2 depicts the processing pipeline we used to extract 3D models of aneurysms from 3DRA images. The exact processing varied slightly for the different data sources, but generally followed the protocol proposed by the @neurIST consortium (11), which puts a special emphasis on the standardization of medical data collection.

**Figure 3:**
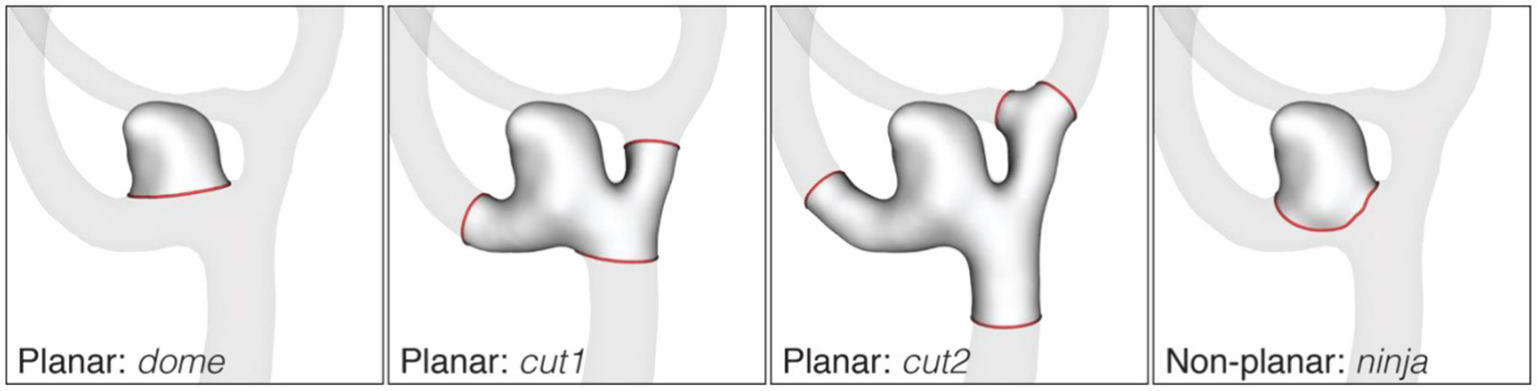
Cut configurations of the AneuX morphology database. Cut lines are shown in red. The *dome* cut disjoins the aneurysm dome from the parent vasculature by one single planar cut. For *cut1* and *cut2*, cut planes are placed perpendicularly to the local centerline in one or two vessel diameters distance from the *dome*. If the rule could not be applied because of an adjacent bifurcation, the closest valid cut before or after the bifurcation was chosen. The non-planar *ninja* cut was placed along the boundary (the so-called neck) of the aneurysmal protrusion. Like the *dome* cut, a *ninja* cut captures the aneurysm dome, but permits a more natural isolation of the aneurysm as assessed by the operator.

The processing of the image data yielded geometric models of the aneurysms, cut free from the parent vasculature in four different configurations as shown in Figure 3: *dome, cut1, cut2, ninja*. Related studies made use of similar dome isolation schemes [*dome*: Ma et al. (16), *cut1*: Berti et al. (11), *ninja*: Mut et al. (17)]. For this processing step, an in-house cut tool based on VTK (18) was used. A final sanitization step ensured similar mesh properties for all 750 geometric models used in this study. Further details about the processing are provided in the Supplementary Material.

### Morphometric Description of the Aneurysms

*Morphological features* or *morphometrics* quantitatively describe the shape of 3D objects, ideally meeting the following requirements: (1) invariance to translation and rotation of the reference coordinate frame, (2) efficiency in encoding relevant morphological information, and (3) robustness with respect to imaging or surface mesh quality. While requirement (1) was satisfied by all morphometric features presented below, one of the purposes for this study was to examine if the candidate features fulfill requirement (2) and (3).

The shape features considered for this study (Table 1) can be grouped into three different categories: *Geometry indices* (GIs) quantify specific geometrical properties of the aneurysm and are typically scalar valued. *Distribution-derived features* include information on the variation of local morphological properties across points (or mesh cells). *Zernike Moment Invariants* are based on a transformed representation of the 2D manifold allowing to derive a set of coefficients capturing the entirety of the geometry under observation.

**Table 1:** Overview of the shape features considered in this study.

**Geometry indices (size)**		**Count: 6; availability: *dome*, *ninja*; references: (16, 19)**
Volume	*V*	Volume of aneurysm dome.
Surface area	*S*	Surface area of the aneurysm dome (without neck area).
Neck diameter	*Dn*	Characteristic diameter of the contour in the neck plane: *D*_*n*_ = 4·*S*_*n*_/*P*_*n*_
Max. diameter	*D*_max_	Diameter of the largest cross-section parallel to the neck plane.
Aneurysm height	*H*	Maximal extent perpendicular to the cut plane.
Aneurysm size	*aSz*	Diameter of the minimum bounding sphere containing the dome.
**Geometry indices (shape)**		**Count: 6; availability:** ***dome***, ***ninja*****; references:** **(****16**, **19****)**
Aspect ratio	*AR*	Ratio between height and neck diameter: *AR* = *H*/*D*_*n*_
Bottleneck factor	*BF*	Ratio between max. diameter and neck diameter: *BF* = *D*_max_/*D*_*n*_
Conicity parameter	*CP*	Measures where the widest cross-section occurs: $CP=0.5-\frac{\;H_{Dmax}\;}{H}$
Non-sphericity index	*NSI*	Measures elongation and undulation; compares the aneurysm to a half-sphere: $NSI=1-{\left(18\pi \right)}^{\frac{1}{3}}\cdot \frac{V^{2/3}\;}{\;S}$. For perfect half-sphere: *NSI* = 0
Ellipticity index	*EI*	Measures elongation; like *NSI*, but evaluated for the dome's convex hull.
Undulation index	*UI*	Measures undulation: *UI* = 1−(*V*/*V*_*CH*_). For convex shapes: *UI* = 0
**Curvature-based indices**		**Count: 8; availability: any cut; references:** **(****16**, **19****)**
Total curvature	*GLN*, *MLN*	Total Gaussian and mean curvature, normalized by surface area.
Total neg. curvature	*NGLN*, *NMLN*	Same as *GLN* and *MLN*, but counting only points with negative curvature. Measures the presence of dents and “saddle-like” regions.
Total curvature normalized by CH	*GLNCH MLNCH*	Total curvature normalized by total curvature of the convex hull. Measures the undulation or blebbiness of an aneurysm.
Entropy of curvature	*GH*, *MH*	Measures how much the curvature varies along the surface (20).
**Writhe-based indices**		**Count: 8; availability: any cut; references:** **(****20****)**
Mean writhe	$W_{mean}^{L_{2}}$	Empirical mean of writhe numbers $W_{i}^{L_{2}}$
Writhe entropy	$W_{H}^{L_{2}}$	Empirical entropy of writhe numbers $W_{i}^{L_{2}}$
Mean writhe, norm.	${\bar{W}}_{mean}^{L_{1}}$	Empirical mean of area-normalized writhe numbers ${\bar{W}}_{i}^{L_{1}}=\;W_{i}^{L_{1}}/\;S$
Writhe entr., norm.	${\bar{W}}_{H}^{L_{1}}$	Empirical entropy of area-normalized writhe numbers ${\bar{W}}_{i}^{L_{1}}$.
**Indices based on Zernike Moments (ZMIs)**		**Count: 126; availability: any cut; references:** **(****21**, **22****)**
ZMIs	$ZMI_{n,l}^{surf}$	Surface-based ZMI, *n* ≤ 20 and *l* such that *n*−*l*>0 and even.
ZMI energy norm.	$Z_{N}^{surf}$	Squared sum of surface-based ZMIs, normalized by fill ratio; evaluated for five different maximum orders *N* = 2, 3, 6, 10, 20.

*Note that the GIs can only be computed for dome and ninja cuts. Details: S_n_, P_n_ are the perimeter and the surface area of the neck orifice. The curvature metrics come in pairs because we considered two types of curvature: Gaussian curvature (symbol: G) and mean curvature (symbol: M). Writhe numbers can be computed with different inner norms (L_1_- and L_2_-norms) and with or without normalization by surface area. ZMIs are identified by two indices: the order n and degree l. The fill ratio is defined as the ratio between the volume of the aneurysm and the volume of the minimal bounding sphere. A total of 150 indices were available for these cut types (12 GIs + 12 DDFs + 126 ZMIs). For cut1 and cut2, a total 138 indices (12 DDFs + 126 ZMIs). CH, convex hull; GIs, geometry indices; DDFs, distribution-derived features; ZMIs, Zernike moment invariants*.

#### Geometry Indices

Geometry indices (GIs) are designed to capture very specific properties of a 3D shape. Advantages of GIs are their geometric interpretability and their low computational complexity. For these reasons, some GIs such as the aneurysm size, neck diameter, or aspect ratio are already routinely measured manually by clinicians. In this study, we used 6 indices for size (dome volume, dome surface area, neck diameter, maximum diameter, aneurysm height, aneurysm size) and 6 indices for shape (aspect ratio, ellipticity index, non-sphericity index, undulation index, conicity parameter, bottleneck factor). These well-established GIs have been previously reviewed by Ma et al. (16), Raghavan et al. (19), and Berkowitz (23). Table 1 provides a summary of these features.

Several metrics require a reference plane at the aneurysm neck. The intersection of this plane with the aneurysm is referred to as *ostium*. For *dome* cuts, this reference plane coincides with the cut-plane. For the non-planar *ninja* cuts, we defined the neck plane as the best-fit plane through the cut line (17).

#### Distribution Derived Features

Distribution-derived features characterize the variation of local shape properties evaluated across points **p** of a surface S. For this study, we considered two such properties, *curvature* and *writhe*, both of which have been used already to characterize IAs (16, 19, 20).

The *curvature* at a point **p** of a surface S can be expressed by means of Gaussian curvature *K*_*G*_(**p**) and mean curvature *K*_*M*_(**p**). We used VTK (18) to compute the local curvature values for discrete surface meshes, which we subsequently aggregated as described in Table 1. A total of 8 different curvature-derived features are evaluated, which include the well-known metrics for total Gaussian and Mean curvature GLN and MLN (16), and two novel metrics.

The *writhe number* measures surface asymmetries and “twisting forces” as seen from a surface point **p** (20). Originally introduced in knot-theory to characterize curves, the writhe number was generalized by Lauric et al. (20) for 3D surfaces. We distinguished between writhe $W^{L_{2}}\left(\text{p}\right)$ and writhe ${\bar{W}}^{L_{1}}\left(\text{p}\right)$ normalized by surface area, resulting in a total of 4 different writhe-based shape features (Table 1). Curvature and writhe features were evaluated for all cut types (*dome, ninja, cut1, cut2*).

#### Zernike Moment Invariants

3D *Zernike moments* (ZMs) and the *Zernike moment invariants* (ZMIs) were first described by Canterakis (24) and applied by Novotni and Klein (25) in the context of 3D shape retrieval. Millán et al. (22) introduced the ZMI for the assessment of intracranial aneurysm morphology.

The goal of 3D Zernike transformation is to describe an input geometry in terms of the so-called Zernike *basis*: a set of (complex-valued) polynomials strongly related to spherical harmonics. The Zernike theory resembles Fourier theory in that a forward transformation yields a set of coefficients (the ZMs) that can be used as weights to reconstruct the original surface through a weighted summation of its basis functions (inverse transformation). The method permits to decompose a geometry into morphological “modes” of gradually increasing complexity. The maximum mode order *n* is chosen so as to capture enough morphological details by the ZMs. ZMs can be made invariant to translation and isotropic scaling (25), but only an additional transformation (the calculation of the Euclidean norm of the ZMs of the same order) yields the rotation-invariant ZMIs, forming a viable shape descriptor.

For this study, we included ZMIs up to order *n* = 20, corresponding to a shape descriptor of 121 independent values. In addition, we introduced the novel metric ZMI *energy* $Z_{N}^{surf}$ (weighted sums-of-squares of ZMIs, divided by the ratio of the aneurysm volume and the volume of the minimal bounding sphere), which we evaluated for five different maximal orders *N* = 3, 6, 10, 15, 20. We focused on *surface-based* ZMIs [as opposed to *volume-based* ZMIs (21, 22)], as they carried a slightly stronger signal in our experiments.

#### Shape Descriptors and Aneurysm Location

We computed the features listed in Table 1 for all 750 aneurysms and all available cut types. We based our analysis primarily on features computed for *dome* cuts unless otherwise noted. Any sequence of one or more morphological features is termed *shape descriptor*.

Motivated by the fact that morphology and the associated risks vary with the anatomical location of IAs (10, 26–28), we added location as the single non-morphometric predictor to our feature pool. The 12 locations considered for this study are listed in **Table 3**. The categorical variable “anatomical location” was represented in the numerical feature space using 12 one-hot-encoded dummy variables, which are all zero, except for the one variable representing the sample's location. The resulting vector containing the dummy variables was used to augment the aneurysm's shape descriptor in experiments that included location as a predictor.

### Benchmarking of Shape Descriptors for Diagnostic Capability

We examined the morphological features for a relation with the aneurysm rupture status. In a first step, we assessed the univariate properties of all morphometric features, and then trained and validated multivariate classification models for the prediction of the aneurysms' rupture status. Our setup was designed as a benchmark to identify feature configurations that have the strongest association with rupture status.

We evaluated unpaired Student's *t*-tests between ruptured and unruptured aneurysms for each of the 150 features. The significance level was set to α = 0.05/*d*, with Bonferroni corrector *d* = 150 to correct for multiple testing (29).

For better comparability of the results, we used the same statistical learning scheme for both univariate and multivariate shape descriptors. All reported results are based on regularized (LASSO) logistic regression models.

#### Preprocessing

We centered and scaled the morphometric features to 0-mean and a standard deviation of 1, which improved convergence rates during classifier training (The submatrix of dummy variables encoding aneurysm location was not standardized). For multivariate models, we optionally reduced the feature space dimensionality by means of a principal component analysis (PCA), selecting the *k* first principal components retaining 90% of the total variance in the (training) data.

#### Model Development and Internal Validation

All logistic regression models were trained and validated using a 5-fold cross-validation (CV) scheme with 20 repetitions, resulting in a total of 100 model evaluations. For training and (internal) validation of the classification models, only HUG data was used. A feasible value for the regularization strength λ, the only tuning parameter of the LASSO cost function, was identified using a grid search. To avoid information leakage between training and test data, the parameters for feature space standardization and optional PCA were computed on training data only.

#### Performance Metrics

For all 100 models trained in this CV setup, we evaluated the ROC-AUC (the area under the receiver operating characteristic (ROC) curve) using the test data and report mean and standard deviation. We further calculated prediction accuracy, sensitivity and specificity at the optimal classifier threshold, characterized by the point on the ROC curve closest to the point (0,1).

#### External Validation

In a final step, we externally validated each of the 100 prediction models using the @neurIST and Aneurisk datasets. Again, we report the average AUC, accuracy, sensitivity and specificity.

#### Feature Space < Configurations

This learning pipeline was applied to all univariate and multivariate models. **Table 4** summarizes the multivariate models considered in this study. Besides the maximal model including all 150 morphometric features (with PCA), a multivariate model was assembled by selecting the best-performers in the univariate model with an *AUC*> 0.7.

### Software Tools

Whilst the implementation of the ZMI was based on C++ code, all other computations were performed in Python 3.6. For the mesh-based operations, we employed VTK [the Visualization Toolkit (18)] and VMTK [the Vascular Modelling Toolkit (30)]. Several utilities to develop, compute and analyze morphometric descriptors have been assembled in our Geometric Modelling Toolkit (GMTK). For the statistical analysis and machine learning, we relied on the Python packages SciPy v1.3 (31), scikit-learn v0.22 (32), and statsmodels v0.11 (33).

## Results

We report here the results for our basic dataset configuration using the two *HUG* datasets consisting of 470 aneurysms (128 ruptured, 342 unruptured, Table 2), and the morphometric features evaluated for the *dome* cut. Statistical learning was performed using LASSO-LR and cross-validation scheme described above, resulting in 100 model evaluations. Deviations from this setup are marked explicitly. The dataset for external validation consisted of 265 cases from the @neurIST and Aneurisk projects. Table 3 summarizes all data used, stratified by aneurysm location and rupture status.

**Table 2:** Summary of the cases included into the AneuX morphology database, stratified by data source.

	**HUG1**	**HUG2**	**@neurIST**	**Aneurisk**	**[-30,-105]116ptOverall**
Number of patients	247	110	151	97	605
Sex	F: 197 (77%) M: 57 (33%)	F: 81 (74%) M: 29 (26%)	F: 109 (67%) M: 42 (33%)	F: 61 (63%) M: 36 (37%)	F: 445 (73%) M: 164 (27%)
Patient age in years (mean ± SD)	F: 56.4 ± 14.0 M: 54.3 ± 13.8	F: 54.4 ± 12.7 M: 50.6 ± 12.2	F: 53.4 ± 12.2 M: 49.8 ± 10.6	F: 53.6 ± 15.2 M: 55.5 ± 10.8	F: 55.0 ± 13.6 M: 52.8 ± 12.2
Number of sIAs	350	135	164	101	750
Ruptured/unruptured	R: 87 (25%) U: 263 (75%)	R: 41 (30%) U: 79 (59%)	R: 89 (54%) U:75 (46%)	R: 44 (44%) U: 57 (56%)	R: 261 (35%) U: 474 (65%)

*Note that for HUG2, the rupture status of 15 aneurysms was not available. SD, standard deviation; sIAs, saccular intracranial aneurysms*.

**Table 3:** Summary of all datasets stratified by aneurysm location and rupture status.

	**HUG1**		**HUG2**		**@neurIST**		**Aneurisk**		**Total**		
**Location**	**U**	**R**	**U**	**R**	**U**	**R**	**U**	**R**	**U**	**R**	**Σ**
MCA bif	57	8	19	4	19	21	14	9	109	42	151
PComA	21	17	9	10	16	38	8	11	54	76	130
AComA	33	43	8	11	0	1	6	17	47	72	119
ICA oph	48	1	16	3	21	5	18	2	103	11	114
ICA bif	15	1	5	0	6	9	2	0	28	10	38
MCA	23	1	5	1	3	0	4	0	35	2	37
BA tip	11	4	4	3	2	7	3	3	20	17	37
ICA cav	28	0	3	0	2	0	1	0	34	0	34
ACA	9	5	5	3	1	3	0	1	15	12	27
VB other	10	2	3	4	1	2	0	1	14	9	23
ICA chor	7	4	2	1	3	2	1	0	13	7	20
PCA	1	1	0	1	1	1	0	0	2	3	5
**Total**	**263**	**87**	**79**	**41**	**75**	**89**	**57**	**44**	**474**	**261**	**735**

*The taxonomy of locations follows Bijlenga et al. (10). U, unruptured; R, ruptured*.

### Univariate Analysis

**Figure 4:**
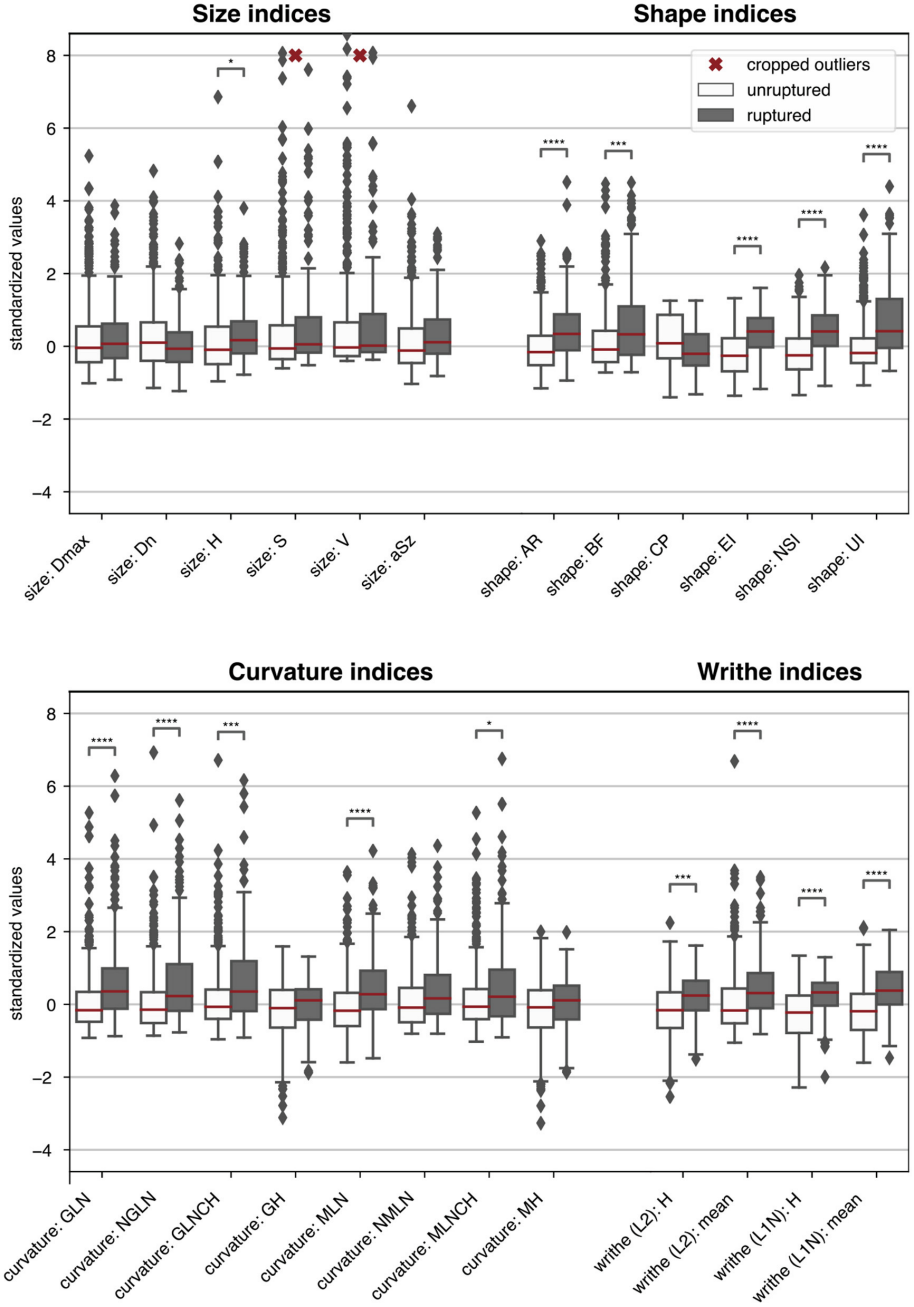
Boxplots summarizing the morphometric data of the 470 HUG samples stratified by rupture status. For easier comparison, each metric was centered and scaled such that the overall median and interquartile range mapped to 0 and 1, respectively. ZMI data was omitted. Single asterisks *, double asterisks **, triple asterisks ***, and quadruple asterisks **** indicate significance for *t*-tests at the α = 0.05, 0.01, 0.001 and 0.0001 level, under consideration of the Bonferroni correction for multiple testing (correction factor 150). The morphometric parameters are described in Table 1.

Figure 4 visualizes the morphometric data on the aneurysms stratified by rupture status (ZMI data was excluded for lack of space). Asterisks indicate if the class differences between the sample means were statistically significant.

**Table 5** presents the predictive accuracy of the 12 best performing features plus aneurysm size (*aSz*). For the sake of brevity, we refer to AUC as the principal comparison accuracy metric. Values for AUC ranged from 0.80 ± 0.06 (for *NSI*) to 0.40 ± 0.08 (for volume *V*).

### Multivariate Analysis

**Table 6** summarizes the internal validation results for the multivariate models from Table 4. For better handling of the high dimensionality of the *MAX* models, PCA was applied, retaining 90% of the total variance present in the data. For the *BUP* models (best univariate performers), we included the features from Table 5. The *LOC* model used only location (Table 3) as predictor and served as reference.

**Table 4:** Description of multivariate models considered in this study and their number *d* of predictors.

**Identifier**	**d**	**PCA**	**Details**
MAX	10	Yes	All morphometric features except for ZMIs of order *n*≥10
MAX+LOC	11	Yes	Same as MAX, extended by anatomical location
BUP	12	No	Independent selection of the best univariate performers with an *AUC*>0.7
BUP+LOC	13	No	Same as BUP, extended by anatomical location
NSI+LOC	2	No	NSI and location
$Z_{6}^{surf}+$LOC	2	No	Normalized ZMI energy for maximum order 6 and location
LOC	1	No	Location only

*BUP refers to the “best univariate performers,” see Table 5. Note that the categorical location predictor expands to 12 (hot-one-encoded) dummy variables*.

**Table 5:** *Internal validation* results of the best univariate classification models, ordered by decreasing ROC-AUC.

**Univariate models (internal validation, cut** ***dome*****)**					
**Category**	**Predictor**	**AUC**	**Accuracy**	**Sensitivity**	**Specificity**
Shape	*NSI*, non-sphericity	**0.80** **±0.05**	0.73 ± 0.04	0.75 ± 0.08	0.72 ± 0.05
ZMI	norm. energy $Z_{6}^{surf}$	**0.80** **±0.05**	0.74 ± 0.04	0.75 ± 0.08	0.74 ± 0.06
ZMI	norm. energy $Z_{3}^{surf}$	**0.78** **±0.04**	0.73 ± 0.04	0.61 ± 0.09	0.78 ± 0.05
Writhe	${\bar{W}}_{mean}^{L_{1}}$	**0.78** **±0.04**	0.72 ± 0.04	0.71 ± 0.09	0.72 ± 0.05
Shape	*UI*, undulation	**0.77** **±0.05**	0.74 ± 0.04	0.61 ± 0.10	0.79 ± 0.05
Curvature	*GLN*	**0.75** **±0.05**	0.71 ± 0.04	0.59 ± 0.08	0.76 ± 0.05
Curvature	*MLN*	**0.75** **±0.05**	0.69 ± 0.04	0.63 ± 0.08	0.71 ± 0.05
Shape	*AR*, aspect ratio	**0.75** **±0.05**	0.70 ± 0.04	0.61 ± 0.11	0.74 ± 0.05
ZMI	$ZMI_{3,1}^{surf}$	**0.74** **±0.05**	0.66 ± 0.04	0.71 ± 0.09	0.64 ± 0.06
ZMI	$ZMI_{5,1}^{surf}$	**0.72** **±0.05**	0.66 ± 0.05	0.68 ± 0.09	0.66 ± 0.06
Writhe	$W_{mean}^{L_{2}}$	**0.72** **±0.05**	0.70 ± 0.04	0.58 ± 0.10	0.74 ± 0.05
Size	*aSz*	**0.64** **±0.05**	0.65 ± 0.04	0.46 ± 0.10	0.72 ± 0.06

**Figure 5:**
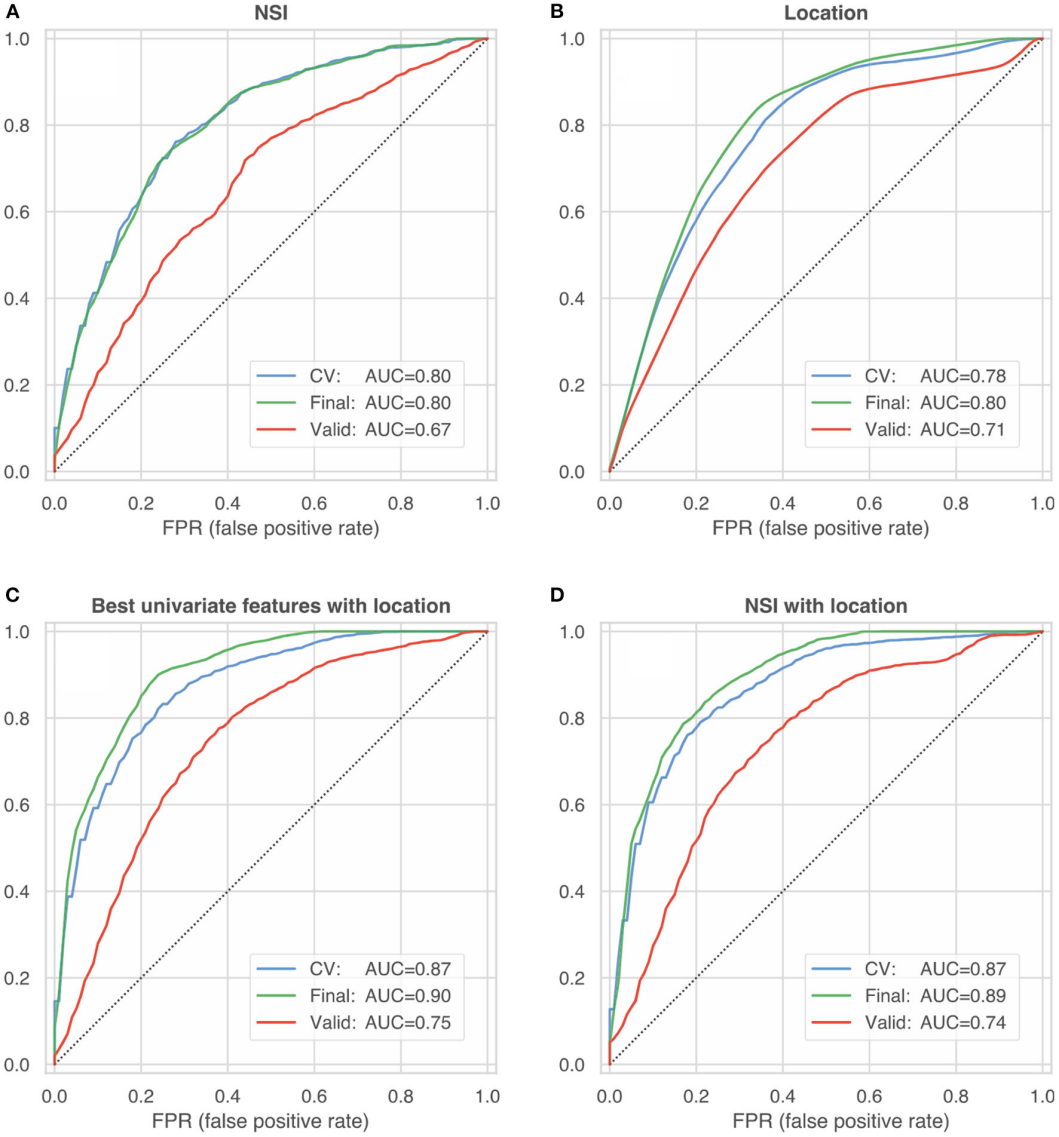
ROC curves summarizing the internal and external validation of four different model configurations: **(A)** non-sphericity NSI **(B)** anatomical location **(C)** best univariate features according to Table 5 with location **(D)** NSI with location. The blue lines represent the *internal* model validation and constitute the mean of 100 ROC curves (computed on test-data folds) during cross-validated training (blue line, CV). The green and red lines characterize the performance of the final model trained on the entire HUG dataset, which was validated on 100 bootstrap samples of the HUG dataset (the training dataset, green lines) and the *external validation* datasets from the @neurIST and Aneurisk projects (red lines).

*We only considered models with an AUC > 0.7 and removed highly correlated features (with a Pearson correlation ρ > 0.95). The list is extended by the best performing size metric: aneurysm size. We report mean and standard deviation (mean ± std) for 100 model evaluations of our cross-validation scheme. The data compares to the blue lines in Figure 5*.

### Validation Using External Data

All univariate and multivariate models were trained and internally validated using HUG data only. After cross-validation based on subsets of training data, final models were computed including all data. These final models were then externally validated using the @neurIST and Aneurisk datasets. We report the resulting metrics of a bootstrapped ROC analysis (with 100 re-samplings of the validation dataset). **Tables 7, 8** summarize the external validation results for the univariate and multivariate models. Figure 5 compares the results with the internal validation using four exemplary models.

### Dependency on the Cut Configuration

We computed the morphometrics for different cut types (Figure 3). Note that the geometry indices (GIs) are defined only for the two cut-configurations solely including the aneurysm dome (planar *dome*, non-planar *ninja*). Features based on curvature, writhe and ZMI were computed on all cuts (*dome, ninja, cut1, cut2*).

Because *dome* and *ninja* cuts both capture the aneurysm dome, the metrics computed for these cut configurations are directly comparable. Some metrics deviated considerably between *dome* and *ninja* cuts. For instance, the aneurysm height *H* varied by up to +50% (for small aneurysms) and +10% in average when going from *dome* to *ninja* cuts. Other metrics also were susceptible to variations in the cut, most notably aspect ratio *AR* (measured as the height-to-neck ratio), the writhe metrics, and the ZMI (with larger differences for higher orders *n*). Aneurysm size *aSz*, *NSI*, and the important curvature metrics *GLN* and *MLN* were comparatively stable. The normalized ZMI energies $Z_{N}^{surf}$ were considerably more stable with respect to alteration in the cutline than the single ZMIs. To summarize the differences $d_{i,j}=x_{i,j}^{dome}-x_{i,j}^{ninia}$ of metric *j* evaluated for the two cut types per (HUG) dataset *i*, we computed the ratio $\delta_{j^{\prime }}$ between median and interquartile range (IQR):


$$\begin{array}{c} \delta_{j^{\prime }}=\delta \left[d_{i,j=j^{\prime }}\right]=\frac{median\left(d_{i,j=j^{\prime }}\right)}{IQR\left(d_{i,j=j^{\prime }}\right)} \end{array}$$

We report here the mean $\bar{\delta }\left[\delta_{j^{\prime }}\right]$ per feature category: GI size ($\bar{\delta }=0.10$), GI shape (δ = 0.21, δ = 0.17 without AR), curvature (δ = 0.17), writhe (δ = 0.22), ZMI (δ = 0.43), ZMI energies (δ = 0.20).

Despite the marked differences between the shape features evaluated for *dome* and *ninja* cuts, their univariate predictive capacity [$AUC\left(x_{i,j}^{dome}\right)$ vs. $AUC\left(x_{i,j}^{ninia}\right)$] was not significantly affected (unpaired *t*-tests, two-sided, α = 0.05, corrected for multiple testing). For the relevant predictors reaching an AUC >0.7 in the univariate models (cf. Table 5), differences in AUC amounted only to fractions of the AUC standard deviation.

Metrics based on cut configurations including segments of the parent vasculature (*cut1, cut2*) generally performed worse than metrics computed for *dome* and *ninja*. Curvature metrics, writhe indices and single ZMIs played no significant role in these experiments (cut1: AUC <0.65, cut2: AUC <0.60). Only the normalized ZMI energies $Z_{N}^{surf}$ maintained their predictive ability, with *Z*_10_ reaching AUC = 0.77±0.06 for *cut1* and AUC = 0.66±0.6 for *cut2*.

## Discussion

Here we examined different aspects of quantitative morphology with the goal to identify shape features that best reflect disease status. With a dataset comprising 470 ruptured and unruptured intracranial aneurysms, we were able to extend several findings from peer literature. We validated the generated univariate and multivariate models against external data provided by the @neurIST and Aneurisk dataset. These findings, as well as the methodological setup *per se*, warrant careful discussion.

In the following, we first comment on the insights from the univariate analysis, mostly focusing on the quantitative description of the aneurysms. We then proceed to compare univariate and multivariate models. Finally, we address some concerns with respect to the methodology, and derive recommendations for future research.

Throughout this discussion, we use ROC-AUC, the area under the receiver operating characteristic curve, as the principal quality metric for diagnostic accuracy of the models. Other metrics such as prediction accuracy, sensitivity and specificity have also been provided (Tables 5–**8**). In our subsequent reasoning we exploit the fact that the training/validation procedures were strictly the same in all experiments, thereby making the results comparable.

### Which Features Encode Disease Status?

#### Geometry Indices

Of all 12 GIs, *NSI* most accurately predicted rupture (AUC = 0.80 ± 0.05). Other shape metrics measuring elongation (*EI*, *AR*) and undulation (*UI*) were also potent univariate predictors for rupture, with AUC scores between 0.75 and 0.79 (Table 5). Metrics capturing the size of the aneurysm were associated with aneurysm rupture (*aSz*: AUC = 0.64 ± 0.05, *H*: AUC = 0.64 ± 0.05), but to a significantly smaller degree than most of the shape metrics (with *BF* and *CP* being the exceptions). The neck diameter *D*_*n*_ (AUC = 0.54 ± 0.06) was not linked to rupture status. These findings underscore the insight that taking into account aspects of morphology other than size can substantially improve the assessment of aneurysms. This lies in contrast with the argumentative line of a previous debate on treatment guidelines, in which size was given more attention than morphology (34, 35).

#### Curvature-Based Metrics

Curvature metrics capture surface undulation and bending. Of all curvature metrics, the well-established *GLN* (AUC = 0.75 ± 0.05) and *MLN* (AUC = 0.75 ± 0.05) performed the best.

#### Writhe-Based Metrics

Writhe-based metrics can be related to surface asymmetry and twisting (20). Our modified definition of surface writhe ${\bar{W}}^{L_{1}}\left(\text{p}\right)$ normalized by the surface area, produced better results than the non-normalized $W^{L_{2}}\left(\text{p}\right)$ (cf. section Distribution Derived Features). Note that ${\bar{W}}^{L_{1}}$ characterizes only shape, whereas the signal contained in non-normalized $W^{L_{2}}$ depends on both shape and size. Indeed, the Pearson correlation coefficient ρ_*P*_ between aneurysm size *aSz* and $W_{mean}^{L_{1}}$ was 0.93; and only 0.20 between *aSz* and ${\bar{W}}_{mean}^{L_{1}}$. Also note that the unmodified definition for surface writhe by Lauric et al. (20) did not prove useful in our experiments (AUC = 0.57 ± 0.06).

#### Zernike Moment Invariants

From 121 considered indices, only the indices $Z_{2,0}^{surf}$, $Z_{3,1}^{surf}$ and $Z_{5,1}^{surf}$ exhibited consistent as well as significant inter-class differences. Higher-order moments yielded either less or no useful information with respect to rupture status. Low order ZMI can be computed with less effort and are more robust with respect to mesh variations than high order ZMI.

#### Normalized Zernike Energies

The $Z_{N}^{surf}$ were good predictors for rupture status. All five ($Z_{1}^{surf},Z_{2}^{surf},Z_{3}^{surf},Z_{6}^{surf},Z_{10}^{surf}$) achieved (univariately) an AUC larger than 0.7. We found that some $Z_{N}^{surf}$ were strongly correlated (Spearman) with undulation/elongation (*NSI*, *EI*, *UI*), *AR* and surface writhe (${\bar{W}}^{L_{1}}$), with correlation coefficients ρ_*Sp*_ between 0.85 and 0.90.

#### Summary

All feature categories except size metrics were well-represented among the best performing candidates (Table 5). Our suggested Zernike energies $Z_{N}^{surf}$ proved to be indicative of the rupture status, which was, along with the non-sphericity index *NSI* (and the highly correlated ellipticity index *EI*), among the best predictors. As far as the distribution-derived metrics are concerned, we recommend using our modified definition of surface writhe ${\bar{W}}^{L_{1}}\left(\text{p}\right)$ and the corresponding index ${\bar{W}}_{mean}^{L_{1}}$. It is worth noting that the metrics that provide good discrimination between ruptured and unruptured aneurysms in this study have been shown to be linked to the clinicians' subjective assessment of the aneurysm shape [perceived shape irregularity (36, 37)].

### How Relevant Is the Cut Configuration?

The cut line separates the aneurysm from the surrounding vasculature and has a bearing on most of the morphometric parameters. To this end, we considered two different cut configurations involving the aneurysm dome only: a planar one (identifier *dome*) and non-planar one (*ninja*). The two sets of rules for separating the aneurysm from the nearby vasculature were applied independently by two operators (one rule for each operator). Naturally, this led to substantial differences in the neck region of the aneurysm geometries (illustrated exemplary in Figure 3).

Albeit these differences, our analysis revealed that the particular choice of the neckline on average had little impact on the metrics' capacity to predict rupture status, indicating that the selected metrics are fairly robust with respect to the cut type. Even though the *ninja* cut has a better physiological justification than the *dome* cut, it did not substantially improve the prediction outcome.

Metrics involving segments of the parent vasculature (*cut1, cut2*) consistently produced worse results compared to *dome* and *ninja* cuts. The more of the parent vasculature was included in the cut, the less accurately the diagnostic models performed (*dome* > *cut1* > *cut2*). The lack of predictiveness in some of the metrics for *cut1*- and *cut2*-geometries does not, however, imply that the parent vessel geometry is irrelevant for disease status prediction. Our collection of features lacks some metrics that relate the parent vessel geometry to the aneurysm dome, for instance size ratio (SR), vessel angle or inclination angle (38, 39).

#### Summary

Both *dome* and *ninja* cuts enabled equal predictive performance of morphometrics.

### Do Combinations of Shape Predictors Lead to a Better Model?

The combination of multiple predictors moderately improved the prediction accuracy (Tables 5, 6), with no signs of excessive model overfitting (green vs. blue lines in Figure 5). However, the net improvement of the multivariate models over the univariate models was relatively small: The best univariate predictor (*NSI*) achieved an AUC of 0.80 ± 0.05.

**Table 6:** *Internal validation* results of the multivariate classification models.

**Multivariate models (internal validation, cut** ***dome*****)**					
**Category**	**#**	**AUC**	**Accuracy**	**Sensitivity**	**Specificity**
MAX (+ PCA)	10*	**0.82** **±0.05**	0.74 ± 0.04	0.75 ± 0.09	0.74 ± 0.04
BUP (best univariate performers)	12	**0.82** **±0.05**	0.74 ± 0.04	0.75 ± 0.08	0.74 ± 0.05
LOC (location only)	12	**0.78** **±0.04**	0.69 ± 0.04	0.78 ± 0.10	0.65 ± 0.05
MAX + LOC (+PCA)	22*	**0.87** **±0.04**	0.79 ± 0.04	0.78 ± 0.08	0.80 ± 0.04
BUP + LOC	24	**0.87** **±0.04**	0.80 ± 0.04	0.77 ± 0.09	0.80 ± 0.05
NSI + LOC	13	**0.87** **±0.04**	0.79 ± 0.04	0.79 ± 0.08	0.79 ± 0.04
$Z_{6}^{surf}$ + LOC	13	**0.87** **±0.04**	0.78 ± 0.04	0.76 ± 0.10	0.79 ± 0.05

Column **# **indicates the dimensionality of the models' feature space, or the (average) number of dimensions retained after PCA if marked with an asterisk. Note that location adds 12 dummy features (one for each location) but represents one single predictor. The data compares to the blue lines in Figure 5. Values marked with an asterisk (*) indicate the number of features after PCA.

The maximal model MAX (AUC = 0.82 ± 0.05) and the BUP model using a selection of best univariate performers (AUC = 0.82 ± 0.05) achieved the same performance. This indicates that the combination of many weak univariate predictors (MAX) does not provide more information about the disease status than a selection of best performers (BUP). This also held true for non-linear classification models: We explored models such as support vector machines with a Gaussian kernel, gradient boosted decision trees and basic neural nets (multilayer perceptrons) (40).

That the MAX and BUP models performed equally well is indicative of redundancy in the descriptors. To assess the level of redundancy, we applied a PCA of the (standardized) feature matrix for the dome cut (470 samples vs. 150 features). A PCA retaining 50, 75, 90, 95, and 99% of the total variance required 5, 19, 44, 62, and 98 of maximally 150 principal components. Some data redundancy could be attributed to the physiological processes that underlie aneurysm formation. For instance, larger aneurysms were more likely to show irregular structures (blebs, lobules), which was also reflected in our data: ρ_*Sp*_(*aSz, GLN*) = 0.82.

Due to these high correlations, we were able to further reduce the number of predictors to four: *NSI*, ${\bar{W}}_{mean}^{L_{1}}$, *GLN* and *aSz*. This reduced model performed about the same as the BUP model: AUC = 0.82 ± 0.04.

#### Summary

Multivariate models (Table 6) performed only slightly better on the HUG dataset than the univariate models, even though the entirety of shape features captured a relatively wide range of morphological characteristics, despite any data redundancy. This corroborates the value of *NSI* and $Z_{N}^{surf}$, but also of ${\bar{W}}_{mean}^{L_{1}}$ and *GLN*, as efficient indicators of those IA shape characteristics that are relevant for distinguishing the rupture status.

#### What Is the Effect of Location as Predictor?

Because aneurysm morphology and associated risks vary significantly with the anatomical location (10), we included location as the only non-morphometric predictor to our models. This resulted in a substantial increase of diagnostic accuracy (AUC =0.87 ± 0.04 for the MAX model and AUC = 0.88 ± 0.04 for the BUP model, Table 6). Two minimal models (*NSI* + location, $Z_{6}^{surf}$ + location) performed both with essentially the same diagnostic accuracy: AUC = 0.87 ± 0.04 (*NSI*) and AUC = 0.87 ± 0.04 ($Z_{6}^{surf}$).

We trained also a location-only model, which performed with an AUC = 0.78 ± 0.04 (Figure 5B). Comparison with the univariate performance of shape predictors (Table 5) suggests that aneurysm location alone is as informative about an aneurysm's rupture status as its morphology.

The probability of rupture varies considerably with location (Table 3). Adding location as a predictor therefore incorporates prior information about the probability of rupture into the classifier.

#### Summary

The addition of aneurysm location improved the predictive accuracy substantially because this enables the classifier to account for varying rupture probabilities. A model relying on *NSI* and location as predictors excelled other models in terms of AUC, prediction accuracy and parsimony.

### External Validation Results

All investigated models performed markedly worse on the external datasets @neurIST and Aneurisk (Tables 7, 8). This indicates that the models do not generalize well to these datasets.

**Table 7:** *External validation* results of the same univariate predictors of Table 5.

**Univariate models (external validation, cut** ***dome*****)**						
**Category**	**Predictor**	**AUC**	**AUC-diff**	**Accuracy**	**Sensitivity**	**Specificity**
Shape	*NSI*, non-sphericity	**0.65** **±0.03**	−0.15	0.62 ± 0.03	0.52 ± 0.04	0.72 ± 0.04
ZMI	norm. energy $Z_{6}^{surf}$	**0.67** **±0.03**	−0.14	0.61 ± 0.03	0.50 ± 0.04	0.73 ± 0.04
ZMI	norm. energy $Z_{3}^{surf}$	**0.70** **±0.03**	−0.08	0.63 ± 0.03	0.47 ± 0.04	0.80 ± 0.03
Writhe	${\bar{W}}_{mean}^{L_{1}}$	**0.69** **±0.03**	−0.09	0.61 ± 0.03	0.52 ± 0.04	0.71 ± 0.04
Shape	*UI*, undulation	**0.66** **±0.03**	−0.11	0.60 ± 0.03	0.44 ± 0.05	0.76 ± 0.03
Curvature	*GLN*	**0.59** **±0.04**	−0.16	0.56 ± 0.03	0.39 ± 0.04	0.73 ± 0.04
Curvature	*MLN*	**0.57** **±0.04**	−0.17	0.54 ± 0.03	0.39 ± 0.04	0.69 ± 0.04
Shape	*AR*, aspect ratio	**0.61** **±0.04**	−0.14	0.57 ± 0.03	0.46 ± 0.05	0.69 ± 0.04
ZMI	$ZMI_{3,1}^{surf}$	**0.71** **±0.03**	−0.03	0.64 ± 0.03	0.64 ± 0.05	0.65 ± 0.04
ZMI	$ZMI_{5,1}^{surf}$	**0.61** **±0.03**	−0.11	0.58 ± 0.03	0.51 ± 0.04	0.65 ± 0.04
Writhe	$W_{mean}^{L_{2}}$	**0.58** **±0.04**	−0.14	0.53 ± 0.04	0.44 ± 0.04	0.61 ± 0.05
Size	*aSz*	**0.50** **±0.04**	−0.14	0.48 ± 0.03	0.36 ± 0.04	0.61 ± 0.04

*The univariate models trained on HUG data were here validated using the @neurIST and Aneurisk datasets. We report mean and standard deviation (mean ± std) for 100 bootstrap samples of the validation data. AUC-diff measures the differences between the AUC scores from the internal validation (Table 5) and the external validation. The data compares to the red lines in Figure 5*.

**Table 8:** *External validation* results of the same multivariate models of Table 6.

**Multivariate models (external validation, cut** ***dome*****)**						
**Category**	**#**	**AUC**	**AUC-diff**	**Accuracy**	**Sensitivity**	**Specificity**
MAX (+ PCA)	10*	**0.67** **±0.03**	−0.15	0.63 ± 0.03	0.66 ± 0.04	0.61 ± 0.04
BUP (best univariate performers)	12	**0.70** **±0.03**	−0.13	0.64 ± 0.03	0.64 ± 0.05	0.64 ± 0.04
LOC (location only)	12	**0.71** **±0.03**	−0.07	0.67 ± 0.03	0.66 ± 0.04	0.69 ± 0.04
MAX + LOC (+ PCA)	22*	**0.73** **±0.03**	−0.14	0.67 ± 0.03	0.62 ± 0.04	0.73 ± 0.04
BUP + LOC	24	**0.74** **±0.03**	−0.13	0.68 ± 0.03	0.59 ± 0.04	0.77 ± 0.03
NSI + LOC	13	**0.74** **±0.03**	−0.13	0.70 ± 0.03	0.62 ± 0.04	0.78 ± 0.04
$Z_{6}^{surf}$ + LOC	13	**0.73** **±0.03**	−0.13	0.68 ± 0.03	0.61 ± 0.05	0.76 ± 0.04

The models were trained on HUG data and validated with @neurIST and Aneurisk data. AUC-diff measures the difference between the AUC scores of the internal (Table 5) and external validation. The data compares to the red lines in Figure 5. Values marked with an asterisk (*) indicate the number of features after PCA.

A closer inspection of the two external datasets revealed several differences that may explain this loss of predictive accuracy. Both @neurIST and Aneurisk datasets exhibited a relatively balanced ratio of ruptured and unruptured cases (Table 3). In total, the validation dataset consisted of 132 unruptured and 133 ruptured aneurysms (1:1), as opposed to 342 unruptured and 128 (3:1) in the HUG training dataset. Furthermore, the distribution of the different locations differed between training and validation datasets. Most notably, the @neurIST dataset comprised only one AComA case, and an equally disproportionate number of PComA cases. Aneurisk matched the HUG datasets in terms of location distribution more closely. However, its unruptured cases were about 50% larger than the average of all unruptured HUG cases (Table 9). Aneurisk's unruptured aneurysms were even larger than the ruptured ones, which was not the case for the HUG datasets.

**Table 9:** Summary statistics for the entire AneuX morpho database, stratified by dataset and rupture status.

		**aSz**		**AR**		**NSI**	
**Dataset**	**#**	**U**	**R**	**U**	**R**	**U**	**R**
**HUG1**	350	5.58 ± 3.98	6.82 ± 3.86	1.01 ± 0.56	1.43 ± 0.77	0.12 ± 0.09	0.20 ± 0.08
**HUG2**	120	5.82 ± 3.07	7.41 ± 4.28	1.03 ± 0.38	1.37 ± 0.56	0.11 ± 0.07	0.21 ± 0.09
**@neurIST**	164	5.93 ± 3.44	6.83 ± 4.08	1.07 ± 0.64	1.33 ± 0.86	0.14 ± 0.11	0.19 ± 0.09
**Aneurisk**	101	8.78 ± 5.47	6.92 ± 4.90	1.28 ± 0.68	1.39 ± 0.57	0.15 ± 0.09	0.19 ± 0.07
**Overall**	735	5.91 ± 4.22	6.93 ± 4.17	1.04 ± 0.56	1.38 ± 0.68	0.13 ± 0.09	0.20 ± 0.09

*We used here median ± IQR because the metrics were not normally distributed. IQR, interquartile range; U/R, unruptured/ruptured; aSz, aneurysm size; AR, aspect ratio; NSI, non-sphericity index*.

All this indicates that the validation dataset (@neurIST + Aneurisk) differed significantly in its structural composition and characteristics from the training dataset (HUG1 + HUG2), with strong repercussions for predictive accuracy. To further substantiate this finding, we repeated the entire analysis using HUG1 as the training and HUG2 as the external validation dataset. Even though HUG1 and HUG2 were processed by different persons, the medical data were collected by the same medical staff in the same period of time, which is likely to have led to a very comparable case selection. This structural data homogeneity in this validation setup translated into substantially improved predictive accuracy, with AUC = 0.84 ± 0.04 for the bivariate model *NSI*+location, AUC = 0.88 ± 0.03 for $Z_{6}^{surf}+$location, and AUC = 0.72 ± 0.05 for the location-only model.

#### Summary

To ensure predictive accuracy, models require that the data they process for prediction possess the same characteristics as the data they have been trained with. However, the HUG datasets and the datasets used for external validation differed in at least three key characteristics: rupture rates, and distributions of size and aneurysm locations.

### Limitations

This study adopts an approach that has recently experienced broader use: Statistical learning schemes are deployed to identify a functional relationship between the quantitative descriptions of aneurysms and a probabilistic assignment of their disease status (19, 20, 22, 38, 41–44). This approach has been criticized, not for the method *per se*, but for the data that are used to train the models (45). In particular, it is doubted whether the insights gained from analyzing the differences between unruptured and ruptured aneurysms can serve as the basis for reliable proxies of “risk” or “instability.” To account for this, we refrained from using such terms in our study and focused on benchmarking the sensitivity of morphological features with respect to the aneurysm's rupture status.

This study is based on data from 3DRA, an angiographic method offering high contrast and resolution when compared with computed tomography angiography (CTA) and magnetic resonance angiography (MRA). Because 3DRA is usually employed only in the context of treatment, datasets that include 3DRAs likely do not adequately reflect the natural distribution of IA characteristics in the general population. While we deem our morphometry benchmarking feasible for 3DRA data in general, we encourage a subsequent study based on MRA and CTA data. In particular, it should be investigated how morphometry depends on image quality (resolution, artifacts).

To assess the generalizability of the findings, it is critical to use multicentric data, as we did in this study. Different selection criteria may apply for different clinical centers, however. Furthermore, characteristics of aneurysm datasets vary over time. For instance, the increased availability of imaging facilities has increased the number of incidentally diagnosed unruptured IAs. As a consequence, the ratio of ruptured to unruptured aneurysms in clinical databases has decreased in recent years. Likewise, the treatment guidelines have evolved, which also affected the selection of cases available for such studies. Trends like these contribute to the above data disparities observed in this study between the HUG, @neurIST, and Aneurisk datasets.

It was conjectured that the morphology of aneurysms might change as a result of rupture (46–48). While this cannot be excluded in general, several studies have suggested that for the majority of ruptured cases this does not apply (19, 27, 35, 49, 50).

A future study could investigate whether the insights of this study remain valid for distinguishing stable and unstable aneurysms (which is clinically more relevant than the ruptured/unruptured dichotomy) and how large the differences must be to detect instability. A dataset based on follow-up data would be very advantageous for a study like the one carried out here. However, as discussed by Ramachandran et al. (51), such datasets can also suffer from selection biases.

## Conclusions

We have conducted a comprehensive study to examine the potency of morphology to encode the disease status of IAs. Based on the AneuX morphology database consisting of 470 aneurysms acquired at the HUG and 265 additional cases from external databases, we investigated how various aspects of the morphometric description of aneurysms relate to rupture status.

Morphology is a good predictor for the aneurysm disease status. Metrics for shape irregularity such as *NSI*, $Z_{N}^{surf}$ and ${\bar{W}}^{L_{1}}$are able to capture relevant shape characteristics to distinguish between ruptured and unruptured cases. In our experiments, these indices performed significantly better than metrics measuring only the size of the aneurysm (e.g., *H* or *aSz*). Quantitative measurement of shape characteristics (especially irregularity) rather than size therefore has the potential to improve the clinical assessment of IAs. The conjunction with aneurysm location favorably affects the predictive power of aneurysm morphology with respect to disease status.

## Data Availability Statement

The raw data supporting the conclusions of this article will be made available by the authors, without undue reservation.

## Ethics Statement

The studies involving human participants were reviewed and approved by the Ethical Committee of the Geneva University Hospitals and by Swissethics (@neurIST protocol, ethics authorization PB_2018-00073, previously CER 07-056). The patients/participants provided their written informed consent to participate in this study.

## Author Contributions

NJ, SS, PB, and SH contributed to conception and design of the study. PB was responsible for the data collection and assisted with data cleaning. NJ organized, processed, and analyzed the data and wrote the manuscript. SS, VK, PB, and SH helped interpret the results. All authors contributed to manuscript revision, read, and approved the submitted version.

## Funding

This work was supported by SystemsX.ch, the Swiss initiative in systems biology, under Grant MRD 2014/261 (AneuX Project) and by the Swiss National Science Foundation under Grant 183'774 (NCCR Kidney.CH).

## Conflict of Interest

The authors declare that the research was conducted in the absence of any commercial or financial relationships that could be construed as a potential conflict of interest.

## Publisher's Note

All claims expressed in this article are solely those of the authors and do not necessarily represent those of their affiliated organizations, or those of the publisher, the editors and the reviewers. Any product that may be evaluated in this article, or claim that may be made by its manufacturer, is not guaranteed or endorsed by the publisher.
