# Supplementary material for: Shape Trumps Size: Image-Based Morphological Analysis Reveals That the 3D Shape Discriminates Intracranial Aneurysm Disease Status Better Than Aneurysm Size
Source: Front Neurol. 2022 May 3;13:809391. doi: 10.3389/fneur.2022.809391 (PMC9110927; doi:10.3389/fneur.2022.809391)
Supplement: Supplementary file 1 [file Data_Sheet_1.pdf]

Supplementary Material

1 Data processing

3D models of aneurysms were extracted from 3DRA images according to the same processing pipeline sketched in Figure 2 of the main article. The exact processing varied slightly for the different data sources, but generally followed the protocol proposed by the @neurIST consortium (1), which puts a special emphasis on the standardization of medical data collection.

In a first step, the data operators extracted 3D surface models of the aneurysm and surrounding vascular segments with the help of either @neuFuse (2) (HUG1, @neurIST) or GIMIAS (3) (HUG2), two highly related software tools developed for this type of problem. The segmentation and surface extraction methods for the Aneurisk data are described in Antiga et al. (4) and Piccinelli et al. (5,6). All methods make use of marching cubes (7) to construct a triangular surface mesh from the binary segmentation images.

In a second step, an operator isolated the aneurysms using planar and non-planar cuts following a set of cut rules (Figure 3 of the main manuscript). The *dome* cut disjoins the aneurysm dome from the parent vasculature by one single planar cut. For *cut1* and *cut2*, cut planes are placed perpendicularly to the local centerline in one or two vessel diameters distance from the *dome*. If the rule could not be applied because of an adjacent bifurcation, the closest valid cut before or after the bifurcation was chosen. The non-planar *ninja* cut was placed along the boundary (the so-called neck) of the aneurysmal protrusion. Like the *dome* cut, a *ninja* cut captures the aneurysm dome, but permits a more natural isolation of the aneurysm as assessed by the operator. Related studies made use of similar isolation schemes (*dome*: Ma et al. (8), *cut1*: Berti et al. (1), *ninja*: Mut et al. (9)). For this processing step, an in-house cut tool based on VTK (10) was used.

To ensure similar mesh properties across different dataset sources and to sanitize the meshes from defects such as minuscule cracks, singular edges or orphaned cells (11) we re-meshed all geometries using VMTK (4). The chosen target cell area of $0.05mm^2$ corresponds roughly to the resolution of typical 3DRAs (with voxel sizes around 300µm). We applied Taubin smoothing (12,13) in preparation for the morphometric description of the aneurysm geometries. We employed the implementation provided by VTK (10) with passband 0.2 and 30 iterations. Note that Taubin's method is a topological smoother that disregards spatial information. The smoothing parameters therefore require readjustment if the mesh resolution varies.

2 References

1. Berti G, Hose R, Marzo A, Villa-Uriol M-C, Singh P, Lawford P. Integrated biomedical informatics for the management of cerebral aneurysms - D23v2 - Analysis Protocols Version 2 [Internet]. Aneurist. 2010. Available from: http://www.aneurist.org/UserFiles/File/PUBLIC_DELIVERABLES/D23v2_v1.2_final.pdf

2. Villa-Uriol MC, Berti G, Hose DR, Marzo A, Chiarini A, Penrose J, et al. @neurIST complex information processing toolchain for the integrated management of cerebral aneurysms. Interface Focus [Internet]. 2011;1(3):308–19. Available from: http://rsfs.royalsocietypublishing.org/cgi/doi/10.1098/rsfs.2010.0033

3. Larrabide I, Omedas P, Martelli Y, Planes X, Nieber M, Moya JA, et al. GIMIAS: An open source framework for efficient development of research tools and clinical prototypes. Lect Notes Comput Sci (including Subser Lect Notes Artif Intell Lect Notes Bioinformatics). 2009;5528:417–26.

4. Antiga L, Piccinelli M, Botti L, Ene-Iordache B, Remuzzi A, Steinman DA. An image-based modeling framework for patient-specific computational hemodynamics. Med Biol Eng Comput. 2008;46(11):1097–112.

5. Piccinelli M, Veneziani A, Steinman DA, Remuzzi A, Antiga L. A framework for geometric analysis of vascular structures: Application to cerebral aneurysms. IEEE Trans Med Imaging. 2009;28(8):1141–55.

6. Piccinelli M, Bacigaluppi S, Boccardi E, Ene-Iordache B, Remuzzi A, Veneziani A, et al. Geometry of the internal carotid artery and recurrent patterns in location, orientation, and rupture status of lateral aneurysms: An image-based computational study. Neurosurgery. 2011;68(5):1270–85.

7. Lorensen WE, Cline HE. Marching Cubes: A High Resolution 3D Surface Construction Algorithm. 1987;21(4):163–9.

8. Ma B, Harbaugh RE, Raghavan ML. Three-dimensional geometrical characterization of cerebral aneurysms. Ann Biomed Eng. 2004;32(2):264–73.

9. Mut F, Löhner R, Chien A, Tateshima S, Viñuela F, Putman C, et al. Computational hemodynamics framework for the analysis of cerebral aneurysms. Int j numer method biomed eng [Internet]. 2011 Jun 1 [cited 2020 May 16];27(6):822–39. Available from: http://doi.wiley.com/10.1002/cnm.1424

10. Schroeder W, Martin K, Lorensen B. The Visualization Toolkit: An Object-Oriented Approach to 3D Graphics. 4th editio. Kitware; 2006.

11. Campen M, Kobbelt L. Practical Guide to Polygon Mesh Repairing. Eurographics [Internet].

2012;(1):1–55. Available from: https://www-i8.informatik.rwth-aachen.de/publication/4/eg2012_tutorial_meshrepair_021.pdf%5Cnhttp://meshrepair.org/eg2012_meshrepair_slides.pdf%5Cnhttps://www.graphics.rwth-aachen.de/publication/4/eg2012_tutorial_meshrepair_021.pdf

12. Taubin G. Curve and surface smoothing without shrinkage. IEEE International Conference on Computer Vision. 1995. p. 852–7.

13. Taubin G, Zhang T, Golub G. Optimal Surface Smoothing as Filter Design. Vol. 16, Teaching Statistics. 1994.
